# The Story of the Insane from Year to Year

**Published:** 1893-11-25

**Authors:** 


### THE STORY OF THE INSANE FROM YEAR TO YEAR.

(Sixth Series.)

### THE INVERNESS DISTRICT ASYLUM.

At the end of the financial year this asylum had the names of 451 patients on its register, showing a decrease of 54 during the twelvemonths. One hundred and sixty-five patients were admitted, 132 recovered, and 40 died, 7 of the deaths being due to phthisis. Dr. Mackenzie mentions the following sad family history when speaking of the readmissions: "She is the mother of one of the women admitted for the third time. Both mother and daughter laboured under recurrent mania, were admitted and discharged during the same week, and on the last occasion were admitted together." The Commissioner in Lunacy begins his report with the following well-merited tribute to the late Medical Superintendent. "It is recorded with regret that since the asylum was last visited Dr. Aitkin, while travelling on the Continent, died after a short illness. He had superintended the asylum since it was opened, and for the long period of thirty years had done his work most faithfully and zealously. He was identified with the whole history of the institution, which had been greatly changed and enlarged while under his care."

### THE LEICESTER BOROUGH ASYLUM.

Here the numbers fell from 517 to 494, but this decrease was unfortunately not due to any lack of lunatics in Leicester, but to the removal of London cases to their own county asylum. During the year 146 were admitted, 40 were discharged recovered, and 27 died. The percentage of recoveries stands at 27·4, and the deaths at 5·6. The latter is extremely low for an English county or borough asylum. As to the causes of insanity in the year's admissions, hereditary influence heads the list with 38, and drink accounts for 17. Dr. Finch had under treatment 99 epileptic patients, and the number of fits registered reached a total of 7,899 during the year. It is always one of our most pleasing duties to chronicle the reception of a pension by an asylum nurse, and we heartily congratulate Nurse Barrow, who, after a service of twenty-two years, received a pension of £30 a year. This may not be very much to live on, but it is always possible to save a little out of wages; and there is now the National Pension Fund, which every asylum nurse ought to join, and so obtain a double pension for her old age. The committee say they "have pleasure in reporting their continued satisfaction with the management of the institution under the care and direction of Dr. Finch." The weekly cost per head is 9s. 5½d.

